# Haemoglobin dynamics in Papuan and non-Papuan adults in northeast Papua, Indonesia, with acute, uncomplicated vivax or falciparum malaria

**DOI:** 10.1186/1475-2875-12-209

**Published:** 2013-06-19

**Authors:** Walter RJ Taylor, Hendra Widjaja, Hasan Basri, Emiliana Tjitra, Colin Ohrt, Taufik Taufik, Samuel Baso, Stephen L Hoffman, Thomas L Richie

**Affiliations:** 1 The US Navy Medical Research Unit no. 2 (NAMRU-2), Jakarta, Indonesia; 2 Department of Tropical Medicine, Tulane University School of Public Health, New Orleans, USA; 3 Centre for Health Research and Development, National Institutes of Health, Jakarta, Indonesia; 4 Armed Forces Research Institute of Medical Science, Bangkok, Thailand; 5 Indonesian Naval Hospital, Jayapura, Papua, Indonesia; 6 Rumah Sakit Umum, Jayapura, Papua, Indonesia; 7 Naval Medical Research Center, Bethesda, MD, USA

**Keywords:** Anaemia, Malaria, Papuans, Indonesia

## Abstract

**Background:**

Haemoglobin (Hb) recovers slowly in malaria and may be influenced by naturally acquired immunity. Hb recovery was compared in malaria immune, indigenous Papuan and non-Papuan adults with limited malaria exposure.

**Methods:**

Hb concentrations were measured on Days (D) 0, 3, 7, and 28 in 57 Papuans and 105 non-Papuans treated with chloroquine, doxycycline or both drugs for acute, uncomplicated *Plasmodium vivax* (n?=?64) or *Plasmodium falciparum* (n?=?98).

**Results:**

Mean (SD, range) D0 Hb was 12.7 (2.2, 7–21.3) g/dL and was similar in *P*. *falciparum* infected Papuans and non-Papuans: 12.2 *vs*. 12.8 g/dL (*P*?=?0.15) but significantly lower in: (i) *P*. *vivax*-infected Papuans *vs*. *P*. *vivax*-infected non-Papuans: 11.4 *vs*. 13.47 g/dL [∆?=?−2.07 (95% CI: –3.3 – –0.8), *P*?=?0.0018], (ii) all patients with splenomegaly (*vs*. those without splenomegaly): 12.16 *vs*. 13.01 g/dL [∆?=?−0.85 (−1.6– –0.085), *P*?=?0.029], and (iii) all females *vs*. all males: 10.18 *vs*. 13.01 g/dL [∆?=?−2.82 (−3.97 – –1.67), *P*?<?0.0001].Multiple regression identified female sex (*P*?=?0.000), longer illness duration (*P*?=?0.015) (*P*. *falciparum* patients) and Papuan ethnicity (*P*?=?0.017) (*P*. *vivax* patients) as significant factors for a lower D0 Hb.

Mean D28 Hb increased to 13.6 g/dL [∆?=?1.01 (0.5-1.5) *vs*. D0 Hb, *P*?=?0.0001]. It was: (i) positively correlated with the D0 Hb (adjusted R^2^?=?0.24, *P*?=?0.000), and was significantly lower in *P*. *vivax* infected Papuans *vs*. non-Papuans: 12.71 *vs*. 14.46 g/dL [∆?=?−1.7 (−2.95– –0.5, *P*?=?0.006).

**Conclusions:**

Haemoglobin recovery was related to baseline Hb. Vivax-infected malaria immune Papuans had persistently lower Hb concentrations compared to non-Papuans with limited malaria exposure. This haematological disadvantage remains unexplained.

## Background

Malaria-associated anaemia is common and occurs in acute symptomatic infections, severe malaria, chronic asymptomatic infections, and inadequately treated or resistant infections [1-3]. Concomitant hookworm infestation, micronutrient deficiency, inherited blood disorders, and HIV compound the anaemic effects of malaria [4,5].

The main pathophysiological mechanisms in malaria related anaemia are the splenic removal of red cells, bone marrow suppression and dyserythropoesis, and acute haemolytic anaemia (AHA) [1,6]. AHA may occur when parasitized red blood cells (PRBCs) are destroyed by the developing parasite and is potentially greater in *Plasmodium falciparum* because red blood cells of all ages are invaded and parasitaemia is high. *Plasmodium vivax* produces a low parasitaemia and invades only reticulocytes [7]. Malaria or drug-induced oxidant stress in patients with glucose-6-phosphate dehydrogenase deficiency may also produce AHA [8,9].

PRBCs and non-parasitized red blood cells (NPRBCs) are removed from the circulation but NPRBCs contribute much more to anaemia and this differs between the species. An estimated 34 NPRBCs in *P*. *vivax*[10] and eight NPRBCs in *P*. *falciparum*[2,11] are removed from the circulation for one PRBC. The splenic threshold for removing red cells is lowered in falciparum malaria [12] and splenic removal is directly related to spleen size [1,13]. Immune related changes to red cells results in their recognition and phagocytosis by activated monocytes and macrophages whilst others are trapped and destroyed in the splenic sinusoids [1,14]. In *P*. *falciparum*, NPRBCs and PRBCs have reduced deformability, making splenic trapping easier whilst, because of their increased size, vivax PRBCs have increased deformability but increased fragility, so splenic destruction is more likely [15-17]. The spleen also removes vivax (K. Chotivanich, unpublished data) and falciparum parasites from red cells and returns the deparasitized red cells back to the circulation, a process called pitting [13,18]; pitted RBCs cells also have reduced survival [19].

Depressed bone marrow function and dyserythropoesis result in reduced red cell genesis and reticulocytaemia and poor bone marrow iron utilization is documented in *P*. *falciparum* despite adequate bone marrow stores [20]. Bone marrow changes may be related to cytokine imbalances in favour of raised TNF [21,22], the toxic effects of haemozoin [23], a blunted response to erythropoietin [24] and relative under-production of erythropoietin in adults [25].

With effective anti-malarial treatment, the mean haemoglobin (Hb) falls initially, reaching a nadir on Day 3 or Day 7 (most studies have collected data on these days only) and rises thereafter to stabilize at six weeks [2,26-28]. Reticulocytes increase and peak after 1 to 2 weeks [24,29], but the bone marrow may still be abnormal after three weeks [1,20].

Naturally acquired immunity to malaria (NAI) plays a role in limiting malaria-associated anaemia. In African children, the prevalence of anaemia, geometric mean parasite densities, and risk of fever with *P*. *falciparum* decrease with increasing age [30,31]. Consistent findings come from northeast Papua where, after approximately two years of intense malaria exposure, malaria naïve non-Papuan adults have similar malariometric indices (malaria prevalence rates, parasite densities, and malaria associated symptoms) as indigenous adult Papuans for *P*. *falciparum* but not for *P*. *vivax*. These data suggest the non-Papuan adults had acquired less protective immunity against *P*. *vivax* compared to *P*. *falciparum*[32,33].

There is a paucity of data on Hb dynamics and malaria-associated anaemia from NE Papua. Herein, a *post hoc* analysis of the Hb changes between malaria-immune, indigenous Papuans and non-Papuans from other parts of Indonesia with limited malaria exposure is reported.

## Methods

The study took place from October 1995 to January 1998 at the Rumah Sakit Umum (RSU), a public hospital in Jayapura, the capital of Papua Province, Indonesia’s most eastern province. Located on the northeast coast of Papua, Jayapura has low rates of malaria transmission but the surrounding lowland area is characterized by intense malaria transmission. Local malariometric data, study conduct and changes in the white blood cell and platelet counts in the same study patients have been detailed elsewhere [34-36]. Briefly, routine haematological parameters (Hb, total white cell and platelet counts) were measured and malaria films taken on Days (D) 0, 3, 7, and 28 during a trial of patients with acute symptomatic, uncomplicated vivax and falciparum malaria who were treated with either chloroquine (C) alone, chloroquine plus doxycycline (CD), or doxycycline (Dox) alone.

Parasite densities (N/μL) were calculated using the measured total white blood cell counts or assumed to be 8000/μL, if the white blood cell count was missing. Double entered and validated data (Epi Info 6.04b, Centers for Disease Control and Prevention, Atlanta, GA, USA) were analysed using Stata v8 (Stata Corporation, USA). Continuous data were compared by ‘t’ test or one way ANOVA or the corresponding non-parametric tests, as appropriate. The relationship between two continuous variables was assessed by Pearson’s correlation coefficient (r) and coefficient of determination (R^2^) for normally distributed data or by Spearman Rho (skewed data). Proportions were compared by Chi-squared test or Fisher’s exact test. Backward stepwise multiple regression was performed to assess the independence of variables on the Hb concentrations; adjusted (a) R^2^ values are reported.

Written, informed consent was obtained from all patients. The study was conducted according to the Indonesian Ministry of Health, the Indonesian Navy, the United States Navy and US Army regulations governing the protection of human subjects.

### Definitions

Anaemia of any degree was defined as Hb concentrations of <13 and?<?12 g/dL in males and females, respectively, and moderate anaemia as Hb?<?11 g/dL for both sexes, adapted from the WHO [37].

**Figure 1:**
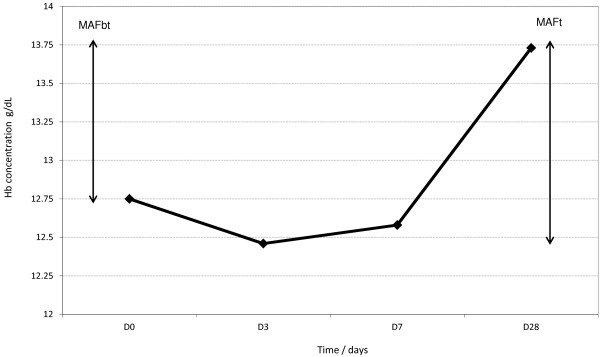
**The mean haemoglobin concentrations at Day 0 and during following up, illustrating the total malaria attributable fall in haemoglobin (MAFt) and the malaria attributable haemoglobin fall before treatment (MAFbt).**

**Figure 2:**
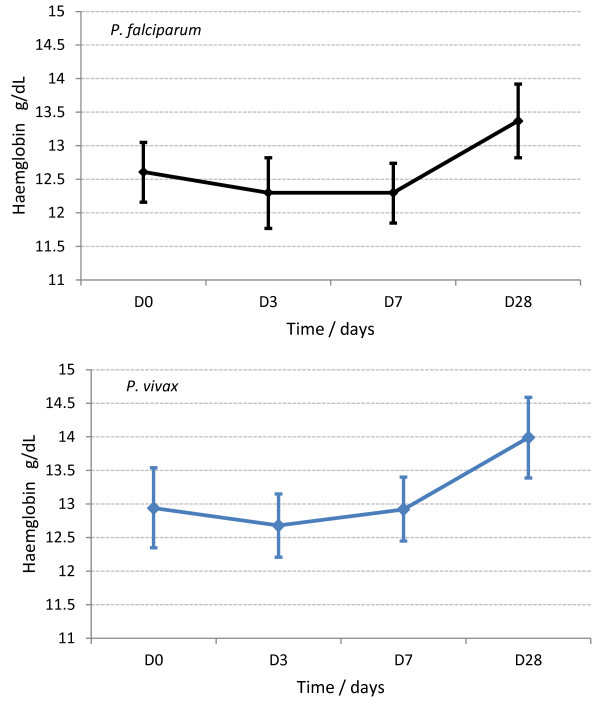
**Haemoglobin dynamics for all falciparum and all the vivax-infected patients.**

The mean total malaria attributable fall in Hb following treatment (MAFt) was defined as the difference between the mean D28 Hb in those successfully treated (i.e. patients without parasites on D28, Hb28wp) and the mean nadir Hb concentration which, in this study, was Day 3 for both species combined (Figure 1) and for each species (Figure 2). The mean malaria attributable fall in Hb before treatment (MAFbt) was defined as the mean Hb28wp minus the mean D0 Hb (Figure 1). The relative MAFt (MAFtrel) was expressed as a percentage of the baseline Hb.

## Results

### General characteristics

A total of 162 adult patients (Papuans?=?57, non-Papuans?=?105) were recruited: *Plasmodium vivax* (n?=?63), *P*. *falciparum* (n?=?89), and mixed infections (n?=?10). These were reclassified as *P*. *falciparum* (n?=?9) and *P*. *vivax* (n?=?1) based on which of the two species had the higher parasitaemia.

Males and females numbered 146 (15–44 years) and 16 (15-33y), respectively. Compared to the non-Papuans, the Papuans had a significantly longer: (i) median residential time, (ii) median time since the preceding episode of clinical malaria, (iii) duration of current illness, (iv) a higher spleen rate (by palpation), (v) a lower median vivax parasitaemia, and (vi) a higher rate of anaemia (Table 1).

**Table 1:** Demographic, clinical and laboratory characteristics of the Papuan and non-Papuan study subjects at disease presentation

	**Papuans**	**Non Papuans**	**P**
	**n=57**	**n=105**	
Age	22 (15–39)	24 (15–44)	0.0028
Male: Female	48 : 9	98 : 7	0.063
Residence in years	21.5 (15–39) y	3 (2m to 24) y	0.000
Previous clinical malaria^*†^	2 (0–16)	3 (0–11)	0.15
Time since last malaria attack^*^	14w (8w-17m)	9w (6w-5.6m)	0.043
Duration of illness in days^†^	4 (1–30)	2 (1–30)	0.0003
Temperature^†^	38 (36.5-41)	38.6 (36.1-41.3)	0.1
Splenomegaly	26 (45.6%)	27 (25.7%)	0.01
Anaemia^§^	34/52 (65.4%)	36/100 (36%)	0.001
Moderate anaemia^|^	16/52 (30.8%)	20/100 (20%)	0.138
*P*. *falciparum* parasitaemia^‡^	3,007(20–64,998)	2,346 (38–74,432)	0.76
*P*. *vivax* parasitaemia^‡^	703 (55–9920)	2,592 (54–14124)	0.034

Continuous data are median (range) unless otherwise stated.

^*^ number of previous malaria episodes by history.

^†^ median (range).

^§^ Anaemia = haemoglobin?<?13 g/dL (male), & 12 g/dL (female).

^|^Moderate anaemia = haemoglobin?<?11 g/dL both sexes.

^‡^ median (range).

### Day 0 haemoglobin

Hb0 data were available for 152 patients. The mean (SD, range) D0 Hb concentration was 12.7 (2.2, 7–21.3) g/dL and was similar between the two species: *P*. *vivax* 12.9 (2.4) g/dL *vs*. *P*. *falciparum* 12.6 (2.1) g/dL (*P*?=?0.35).

Significantly lower mean D0 Hb concentrations (Table 2) were found in: (i) vivax-infected Papuans *vs*. non-Papuans, (ii) all patients with splenomegaly (n?=?47): 12.16 *vs*. 13.01 g/dL (without splenomegaly, n?=?105): ∆?=?−0.85 (−1.6 – –0.085), *P*?=?0.029, and (iii) all females (n?=?14) 10.18 *vs*. 13.01 g/dL (males, n?=?138): [∆?=?−2.82 (−3.97 – –1.67), *P*?<?0.0001.

**Table 2:** **Mean haemoglobin (standard deviation) and mean haemoglobin changes at follow up in Papuan and non-Papuan adults following treatment for *Plasmodium vivax *or *P*. *falciparum *malaria**

	**Papuans**	**N**	**Non Papuans**	**N**	**P**
*Day 0*					
Hb	11.85 (1.88)	57	13.5 (2.27)	105	0.009
Males	12.27 (0.26)	44	13.35 (0.22)	94	0.005
Hb falciparum	12.16 (1.97)	36	12.94 (2.18)	53	0.1
Hb vivax	11.4 (1.59)	16	13.47 (2.35)	47	0.001
*Day 3*					
Hb	11.93 (2.02)	46	12.86 (1.73)	90	0.006
Hb falciparum	12.14 (2.12)	31	12.65 (1.83)	47	0.26
Hb vivax	11.5 (1.78)	15	13.09 (1.61)	43	0.0024
∆ Hb3 Hb0	−0.03 (−0.31 to?+?0.24)	46	−0.27 (−0.63 to?+?0.09)	90	0.39
∆ Hb3 Hb0 falciparum	– 0.11 (−0.48 to?+?0.25)	31	−0.17 (−0.6 to?+?0.26)	47	0.85
∆ Hb3 Hb0 vivax	0.12 (−0.28 to?+?0.53)	15	−0.38 (−0.99 to?+?0.29)	43	0.33
*Day 7*					
Hb	12.2 (1.95)	43	12.8 (1.76)	83	0.1
Hb falciparum	12.28 (2.07)	29	12.3 (1.71)	40	0.96
Hb vivax	12.06 (1.75)	14	13.21 (1.71)	43	0.035
∆ Hb7 Hb0	0.31 (−0.05 to?+?0.68)	42	−0.23 (−0.71 to?+?0.12)	83	0.059
∆ Hb7 Hb0 falciparum	0.17 (−0.28 to?+?0.64)	28	−0.44 (−0.09 to +0.96)	40	0.09
∆ Hb7 Hb0 vivax	0.56 (−0.07 to?+?1.26)	14	−0.17 (−0.49 to +0.82)	43	0.2
*Day 28*					
Hb	13.07 (2.03)	27	13.9 (1.49)	47	0.038
Hb falciparum	13.23 (2.15)	19	13.49 (1.54)	25	0.64
Hb vivax	12.71 (1.79)	8	14.46 (1.28)	22	0.006
∆ Hb28 Hb0	1.15 (0.55 to 1.76)	26	0.94 (0.25 to 1.63)	47	0.66
∆ Hb28 Hb0 falciparum	1.06 (0.23 to 1.9)	18	1.2 (0.3 to 2.1)	25	0.82
∆ Hb28 Hb0 vivax	1.36 (0.46 to 2.26)	8	0.64 (−0.48 to 1.75)	22	0.44

There was a weak negative relationship between illness duration and D0 Hb (R^2^?=?0.04) and no correlation between the D0 Hb and parasitaemias of both species (*P*?=?0.77, *P*?=?0.54). Multiple regression of the falciparum-infected patients identified female sex (*P*?=?0.000) and illness duration (*P*?=?0.015) as significant factors for a lower D0 Hb. In the vivax-infected patients, being Papuan (*P*?=?0.017) was the only significant variable; it was also associated with a lower D0 Hb.

### Haemoglobin dynamics

**Figure 3:**
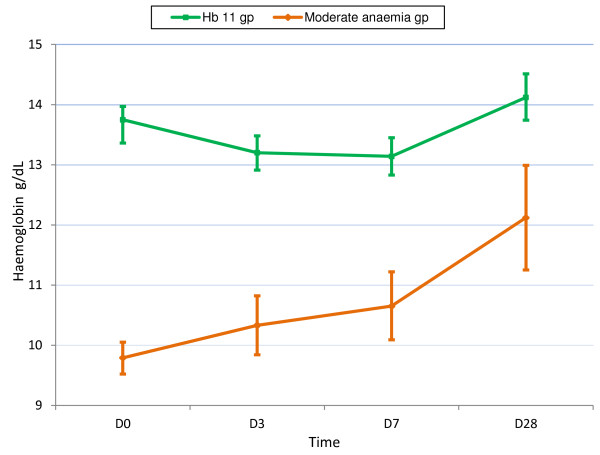
Haemoglobin dynamics for all patients combined as a function of baseline moderate anaemia (Hb <11 g/dL).

For all patients combined, the mean Hb concentration fell to nadir on Day 3: -0.19 (−9.4 - 4.2) g/dL [≡ −0.55 (−44.1 - 44.7) %]; this fall was inversely related to baseline Hb (*P*?=?0.000, Additional file 1). By contrast, patients with baseline moderate anaemia had an initial rise on mean Hb (0.58 (−1.2 – 4.2) g/dL on D3) that was sustained to D28 (Figure 3).

**Figure 4:**
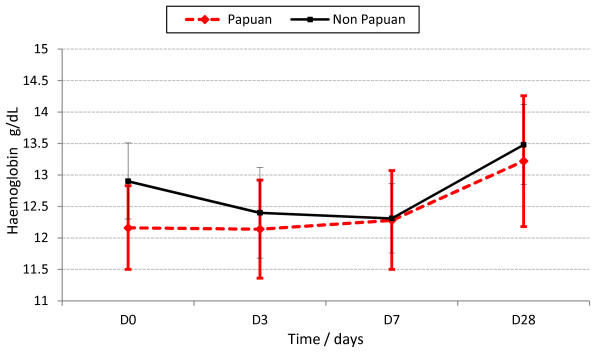
**Haemoglobin dynamics in the falciparum infected Papuans and non-Papuans.**

**Figure 5:**
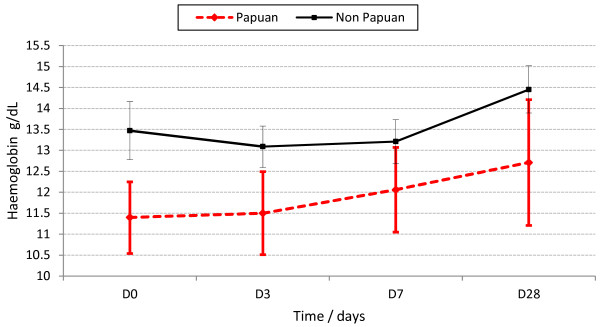
**Haemoglobin dynamics in the vivax-infected Papuans and non-Papuans.** The mean haemoglobin concentrations were significantly lower in the Papuans at each time point (*P*?≤?0.035), despite overlapping 95% confidence intervals.

For all patients combined, Hb dynamics were similar between the species (Figure 2), the falciparum infected Papuans (Figure 4), and the three drug arms (Additional file 2). The vivax-infected Papuans had a small mean rise in Hb by D3 (not significantly different to the small increase in the non-Papuans, *P*?=?0.33) but had significantly lower mean Hb concentrations on D3-28 compared to the non-Papuans with *P*. *vivax* (Table 2 and Figure 5).

By D28, the mean Hb (n?=?74 patients) had risen significantly (*P*?=?0.0001) to 13.6 g/dL and there was a corresponding fall (*P*?<?0.001) in the proportion of anaemic patients to 29.73% (22/74) *vs*. baseline. The D28 Hb concentration was best explained by the D0 Hb concentration and sex (multiple regression): aR^2^?=?0.32 (*P*?=?0.001); removing sex from the model resulted in an aR^2^?=?0.24 for D0 Hb.

### Malaria attributable fall in haemoglobin

On Day 28, there were 65 patients who were parasite negative and had paired Hb3 and Hb28 data; their mean D28 Hb was 13.74 (SD 1.7, range 9.9-18.1) g/dL. The mean (range) MAFt and MAFtrel values were 1.41 (−1 - 5.6) g/dL and 12.3 (−8.5 - 62) %, respectively. The mean MAFbt was 1.07 (−7.9 -6.2) g/dL, representing 75.8% (1.07/1.41) of the MAFt. MAFt and MAFtrel gave essentially the same results in all analyses so MAFtrel results are reported hereafter.

In bivariate analyses, MAFtrel was significantly: (i) negatively correlated with the D0 Hb (Pearson’s coefficient?=?−2.8, *P*?=?0.0001), (ii) positively correlated with illness duration (*P*?=?0.0005), (iii) higher (*P*?=?0.0005) in patients with D0 moderate anaemia (n?=?16) *vs*. those without moderate anaemia (n?=?49): 23.3% *vs*. 6.6% (these %s correspond to MAFt concentrations of 1.9 *vs*. 0.9 g/L, (iv) higher (*P*?=?0.0067) in patients with splenomegaly (n?=?24) *vs*. those without splenomegaly (n?=?41): 18.2% *vs*. 8.8% (MAFt concentrations of 2.03 *vs*. 1.05 g/L).

Non-significant MAFtrel factors included: (i) ethnicity: Papuan 10.9% *vs*. non-Papuan 13.1% (*P*?=?0.53), (ii) drug treatment (*P*?=?0.53): 7.3% (C) *vs*. 12.6% (CD) *vs*. 13.7% (Dox), and sex (*P*?=?0.36): 16.8% (female) *vs*. 11.7% (male).

Multivariate analysis in the falciparum infected patients (aR^2^?=?0.47), identified four significant variables: Papuan ethnicity and D0 Hb were inversely correlated whilst illness duration and splenomegaly were positively correlated (Table 3). There were no explanatory variables in vivax patients (aR^2^?=?0).

**Table 3:** Multivariate analyses showing significant factors associated with the total malaria attributable fraction relative to the baseline haemoglobin in falciparum infected patients

**Independent variable**	**Coefficient**	**95% CIs**	**P**
Papuan ethnicity^*^	−6.41	−12.73 – –0.099	0.047
Day 0 haemoglobin^†^	−3.37	−4.95 – –1.79	0.000
Day 0 splenomegaly^*^	6.69	0.17 – 13.21	0.045
Illness duration^§^	0.71	0.073 – 1.34	0.030
Constant	49.53	29.02 – 70.04	0.000

No factors were identified in the vivax infected patients.

^*^ dichotomous variable (present yes / no).

^†^ Continuous variable measured in g/dL.

^§^ measured in days.

## Discussion

This study has shown that, for all patients and when stratified by infecting species, haematological recovery was characterized by an initial fall in the mean Hb, followed by a rise, consistent with previous studies of falciparum and vivax malaria [2,38]. By contrast, patients with moderate anaemia had an initial rise in mean Hb, suggesting they had longer illness durations, had reached their nadir Hb at presentation and were “primed” to respond to anti-malarial treatment.

For all patients, the initial fall in mean Hb was modest, ~0.2 g/dL (~0.5% *vs*. D0 Hb), and was inversely related to the baseline Hb. The subsequent increase in mean Hb, measured by the MAFt, was 1.4 g/dL; this figure compares favourably to the ~1.2 g/dL and ~1.1 g/dL in falciparum-infected patients of all ages from western Thailand [2] and Laotian adults [28], respectively, whose follow up was longer (42 days).

The MAFtrel was used in these analyses as it might be better than the MAFt as marker of Hb recovery because it takes into account the baseline Hb. No independent factors were identified to explain the MAFtrel in the vivax patients, suggesting inadequate patient numbers. For the falciparum patients, a higher MAFtrel was associated with a lower D0 Hb, a longer illness duration and splenomegaly whereas a lower MAFtrel was associated with being Papuan; these four factors accounted for just under half of the variance of the MAFtrel. Interestingly, ethnicity was not significant in the bivariate analysis and when excluded from the multivariate analysis, the model was less good. Given that the falciparum infected Papuans had similar Hb dynamics as the non-Papuans; these statistical findings regarding the Papuans should be interpreted cautiously.

Longer illness duration and splenomegaly are factors associated with a lower D0 Hb which in turn is a stimulus for increased erythropoietin production. Thus, with treatment, the bone marrow response may be more robust as the suppressing effects of cytokines and haemozoin on it are reduced and as spleen size reduces, so red cell survival increases [1,22-24]. Although the bone marrow response appears more robust in patents with a lower baseline Hb, they did not “catch up” with the non-anaemic patients by study end. Furthermore, the D28 and D0 Hbs correlated positively (the latter explained ~25% of the D28 Hb variance). Therefore, “if you start low, you finish low”, despite better MAFtrel values. Longer follow up would have given data on recovery times for the anaemic patients and may have shown, like Price *et al*. in Karen children and adults, that anaemic patient recover more slowly than those without anaemia [2]. Moderate anaemia (<11 g/dL) was common (~23%) at presentation and is in broad agreement with 18% found in the Karen, using a haematocrit <30% [2]. Factors associated with anaemia in the Karen were with age <5, splenomegaly, hepatomegaly, female sex, and prolonged illness, overlapping with some of the findings in the Papuan and non-Papuan adults.

The roles of ethnicity and NAI in the epidemiological context of NE Papuan are interesting questions. Data from this study suggest that the Papuans were haematologically disadvantaged when infected with *P*. *vivax*; they had persistently lower mean Hb concentrations post treatment compared to the non-Papuans. The indigenous Papuans had greater malaria exposure, because of their longer residence in Papua, suggesting that there may have been differences in NAI between the two groups. Data from this area show there is divergence in the acquisition of NAI for the two main malaria species. NAI to *P*. *falciparum* develops after approximately two years of intense transmission in newly arriving non-Papuans [32,33]. The median residence time for non-Papuans in this study was 3 years; thus, half or more of them probably had similar degrees of NAI to *P*. *falciparum* as the Papuans. Similar degrees of NAI against *P*. *falciparum* probably explain the similar baseline parasitaemia and mean Hb concentrations from baseline to D28 despite the initial fall in mean Hb for the non-Papuans. By contrast, NAI to *P*. *vivax* takes longer to acquire and NAI was higher in the Papuans as evidenced by their significantly lower median, baseline vivax parasitaemia; however, their vivax related NAI was not haematologically beneficial.

The Papuan haematological conundrum is unexplained and might represent host factors like G6PD deficiency, ovalocytosis, HIV, iron deficiency or pathophysiological factor/s unique to the Papuans that operate more in *P*. *vivax* infections, e.g. greater red cell fragility/splenic destruction, more cytokine induced bone marrow suppression, slower bone marrow recovery. These speculations underscore the lack of knowledge of the mechanisms of Hb dynamics in these two populations and call for further detailed investigation [39].

This study has several limitations. The analyses were supplementary to those of a small clinical trial of 162 adult patients and follow up was 28 days, two weeks before Hb stabilizes. By contrast, Price *et al*. analysed ~1,500 adults and children over 63 days [2]. The study herein reported was not designed *a priori* to detail the haematological responses; thus, key haematological parameters like the mean corpuscular volume, reticulocyte counts, iron metabolic parameters, G6PD status, presence of thalassaemia and ovalocytosis were not tested. Such data would be essential in a haematological investigation in ethnically diverse NE Papua.

To conclude, this study has shown similar Hb dynamics between vivax and falciparum malaria but un explained differences between vivax-infected Papuans and non-Papuans.

## Competing interest

None of the authors have a conflict of interest.

## Authors’ contributions

CO conceived the original design. CO, WRJT, ET, SLH, TLR developed the protocol. WRJT, HW, HB, Taufik, ET, SB executed the study. WRJT analysed the data and wrote the first draft of the paper. TLR, SLH, CO, ET contributed significantly to revisions of the paper. All authors read and approved the final manuscript.

## Supplementary Material

Additional file 1 The relationship between the absolute change in the initial fall (hbdiff30) in haemoglobin on Day 3 and the baseline haemoglobin concentration (hb0) in g/dL for the Papuans and non-Papuans combined. Click here for file

Additional file 2 **Box plots of haemoglobin dynamics as a function of treatment arms in all patients with both species.** C = chloroquine alone, CD = chloroquine plus doxycycline, Dox = doxycycline alone. Click here for file
