# Supplementary figures and images for: Haemoglobin dynamics in Papuan and non-Papuan adults in northeast Papua, Indonesia, with acute, uncomplicated vivax or falciparum malaria
Source: Malar J. 2013 Jun 19;12:209. doi: 10.1186/1475-2875-12-209 (PMC3691772; doi:10.1186/1475-2875-12-209)

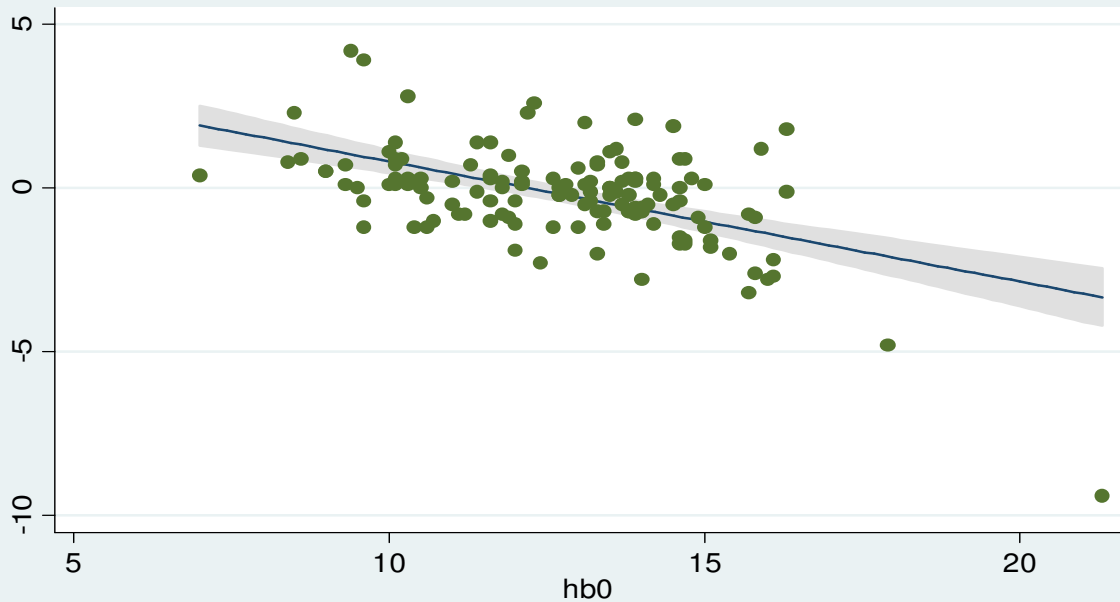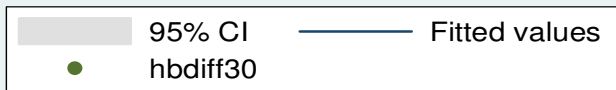

Supplement: Additional file 1 — The relationship between the absolute change in the initial fall (hbdiff30) in haemoglobin on Day 3 and the baseline haemoglobin concentration (hb0) in g/dL for the Papuans and non-Papuans combined. [file 1475-2875-12-209-S1.pdf]

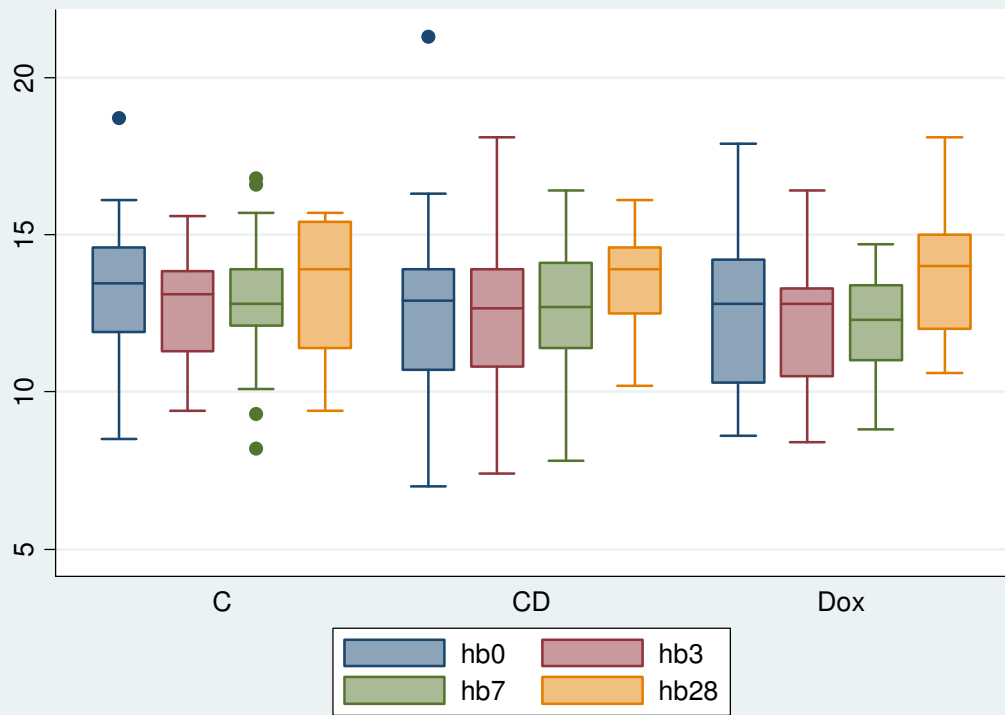

Supplement: Additional file 2 — Box plots of haemoglobin dynamics as a function of treatment arms in all patients with both species. C = chloroquine alone, CD = chloroquine plus doxycycline, Dox = doxycycline alone. [file 1475-2875-12-209-S2.pdf]
